# Hospital Festivals and Meetings

**Published:** 1894-10-20

**Authors:** 


### HOSPITAL FESTIVALS AND MEETINGS.

### CHELSEA HOSPITAL FOR WOMEN.

A special meeting of the Governors of the Chelsea Hospital for Women was held on Wednesday, October 10th, 1894, to receive a statement from the Board of Management as to the carrying out of the recommendations of the late Commission of Inquiry, and to receive the resignations of the Chairman and the other members of the Board of Management. In the absence of Lord Cadogan and Mr. Wright Biddulph, the chair was taken by the Vice-Chairman of the Board of Management, Mr. Thomas W. Brooks.

The statement read dealt with the recommendations of the Committee of Inquiry under three main heads—sanitary, medical, and management. With regard to the first of these, in the report of the Committee of Inquiry it was stated that since the time of Dr. Parkes' comments upon the defective sanitary condition of the hospital, the hospital authorities have "thoroughly overhauled the whole of the sanitary arrangements of the building, and it is reported to us that they have been put into as complete a sanitary condition as is possible at the present time." The Board now "at this later period report that, so far as human ingenuity and outlay can secure the sanitary perfection of the building, the hospital may now be said to be perfect." The Board considered that everything possible to be done was done when the hospital was built twelve years ago, but the advances in sanitary science since then had been great, and what is perfect to-day may be imperfect to-morrow. All the suggestions of the commission had been unhesitatingly carried out except in the case of Dr. Parkes' proposal to divert the main drain from the outside to where it now is, under the centre of the building, but compulsion from Dr. Parkes and the Vestry proving too strong, this change has also been effected. It was now too late to refer to some of the angry and sensational statements which had been made with regard to the hospital, but the Board thought it was not too late to remind the governors that Dr. Parkes went through the farce of taking steps to close the hospital when it had already been closed for three weeks by the Board themselves, and that they had obtained the Commission of Inquiry when Dr. Parkes and the Vestry failed to do so. Dr. Parkes' method of doing his duty might have wrecked a worthy medical charity had not the governors and subscribers rallied to its support. The money spent upon the complete renovation of the hospital amounted to a sum of £1,251 3s. 8d.

The medical arrangements occupied the chief and most important part of the report of the commission. As soon as the report was published the members of the medical staff resigned in a body. The Board proceeded to fill the vacancies, for which there were 34 candidates. A committee to consider the applications was appointed by the Board of three members of the consulting staff and three lay members of the Board, and each candidate was invited to attend before the committee. The committee deemed it advisable to recomment to the Board that two in-patient and four out-patient physicians only should be appointed for the moment, and sent up the names of four candidates for the former and eight for the latter office. These applications were considered at a full meeting of the Board, with Lord Cadogan in the chair, and the following were elected: Dr. W. Duncan, Dr. Fenton, Dr. Inglis Parsons, Mr. O'Callaghan, Dr. Giles, and Dr. Eden. Subsequently the consulting physicians resigned, and their resignations were accepted with much regret. It was proposed to elect to this important office eminent consultants in the speciality of the hospital, who were in actual consulting practice, and occupying similar positions at other hospitals, so that the medical staff might have the advantage of their active help in all cases of difficulty.

On the third point dealt with, that of the management of the hospital, the Commission of Inquiry used these words, "We are of opinion that owing to want of system, there has not been proper control of the medical staff by the managers of the hospital." The Board proposed to ask for powers "to suspend or dismiss any member of the medical staff, after full inquiry, if he has acted in a manner detrimental to the interests of the hospital." It was also proposed that the medical staff should retire annually as do the Board of Management, but be eligible for re-appointment. The medical staff had been constituted a medical committee to advise the Board on all professional matters. A pathologist, to be directly responsible to the Board, had yet to be appointed. In the case of any death occurring in the hospital a special report would now be required from the medical officer attending the case. The beds in the hospital had been reduced from 60 to 42 to allow of the increased cubical area for each bed as suggested by the Commission. For some time the Board of Management had been meeting weekly, but it was now proposed that they should meet monthly in future, as heretofore, and that a weekly Board should be appointed, to meet weekly, with a separate chairman, neither the Board of Management nor the weekly Board to include any medical men. Sir Henry Calcraft had been kind enough to join the Board of Management, which would benefit much by his great experience. To conclude, the Board now felt that having stuck to the ship through its recent troubles, and having accomplished certain reformations, the time had arrived when they might with dignity place their resignations in the hands of the governors, to be re-elected or not, as might be considered best for the interests of the hospital.

A somewhat heated discussion followed, the action of the Board with regard to the appointment of the new medical staff being objected to by Dr. Travers, Dr. Schacht, the Rev. Mr. Myers, and others. Dr. Travers said he held letters from Dr. Robert Barnes, Sir Spencer Wells, and Mr. Hutchinson, in which they expressed their disapproval of the mode in which the elections had been made. In replying the Chairman took exception to certain circulars issued to the out-patients by Dr. Schacht, stating that he would see them at his own house in future, his connection with the hospital being at an end. After a temperate speech from Mr. R. Chapman, and a few words from the Rev. Prebendary Webb-Peploe, disclaiming any intention on the part of the Board to reflect discredit upon those members of the late medical staff who had not been re-elected, Dr. Schacht's resolution that the resignation of the Board of Management be accepted was put to the meeting, and was lost by fourteen votes to eight, some of the governors, of whom between thirty and forty were present, not voting at all. The Board of Management remains, therefore, constituted as before.

